# Accuracy of Chatbots in Citing Journal Articles

**DOI:** 10.1001/jamanetworkopen.2023.27647

**Published:** 2023-08-08

**Authors:** Anjun Chen, Drake O. Chen

**Affiliations:** 1 Learning Health Community, Palo Alto, California; 2 ELHS Institute, Inc, Palo Alto, California

## Abstract

This cross-sectional study quantifies the journal article citation error rate of an artificial intelligence chatbot.

## Introduction

The recently released generative pretrained transformer chatbot ChatGPT from OpenAI has shown unprecedented capabilities ranging from answering questions to composing new content.^1^ Its potential applications in health care and education are being explored^2^ and debated.^3^ Researchers and students may use it as a copilot in research. It excels at creating new content but falls short in providing scientific references. Journals such as *Science* have banned chatbot-generated text in their published reports.^4^ However, the accuracy of reference citing by ChatGPT is unclear; therefore, this investigation aimed to quantify ChatGTP’s citation error rate.

## Methods

This study tested the value of the ChatGPT copilot in creating content for training of learning health systems (LHS).^5^ A large range of LHS topics were discussed with the latest GPT-4 model from OpenAI from April 20 to May 6, 2023. We used prompts for broad topics, such as LHS and data, as well as specific topics, such as building a stroke risk prediction model using the XGBoost library (Table 1). Since chatbot responses depended on the prompts, we first asked questions about specific LHS topics, then requested journal articles as references. This study followed the Strengthening the Reporting of Observational Studies in Epidemiology (STROBE) reporting guideline.

**Table 1.** Examples of a Sequence of Prompts to Engage GPT Chatbots for Discussing LHS Topics

Topic^a^	Order^b^	Prompt^c^
LHS	1	LHS vision will transform our health care systems. What is LHS? Provide some journal articles for LHS as reference.
Clinical study	2	LHS embeds clinical research in care delivery. Provide some journal articles for embedded clinical studies.
Clinical study	3	In LHS, I can conduct observational studies. Give me 10 journal articles on observational studies.
Data	4	LHS uses terminology standards for patient data. Provide 10 journal articles on medical terminology standards.
Data	5	UMLS integrates all standard vocabularies. Please provide 10 journal articles on UMLS standard.
ML	6	ML can build risk prediction models. Provide 10 journal articles for machine learning risk prediction models.
ML	7	XGBoost is a common ML algorithm. Provide 10 journal articles for XGBoost risk prediction models.
ML	8	ML can use EHR data. Provide 8 journal articles for stroke risk prediction models using EHR data.
ML	9	Deploying ML model is challenging, right? Give me some journal articles about deployment of risk prediction ML models.
Regulation	10	Deploying ML models in health care is regulated. Do you have some journal articles about regulation of using risk prediction ML or AI models in clinical settings.

Abbreviations: AI, artificial intelligence; EHR, electronic health record; LHS, learning health system; ML, machine learning; UMLS, unified medical language system.

^a^ Examples of broad topics included health systems; clinical trials; health care delivery; LHS vision, progress, and challenges; government policies and fundings; LHS education and training; medical knowledge and evidence generation; health data and EHRs; synthetic data and simulation; terminology, ontology, data model standards, and interoperability; ML and knowledge graph; deployment and generalizability; and clinical research network. Examples of narrower topics included stroke risk prediction model built by XGBoost and patient risk factor graph integrated with UMLS knowledge subgraph.

^b^ Indicates order of prompts (or questions).

^c^ Prompt should provide context, ask topic questions, and then request for references.

We verified each cited journal article by checking its existence in the cited journal and by searching its title using Google Scholar. The article’s title, authors, publication year, volume, issue, and pages were compared. Any article that failed this verification was considered fake. To determine a reliable error rate, over 300 article references were produced on the LHS topics. For comparison, we chatted with OpenAI’s default GPT-3.5 model for the same LHS topics. Exact 95% CIs for error rate were constructed. The error rate between the GPT-4 and GPT-3.5 models was compared using the Fisher exact test, with 2-sided *P* < .05 indicating statistical significance.

## Results

From the default GPT-3.5 model, 162 reference journal articles were fact-checked, 159 (98.1% [95% CI, 94.7%-99.6%]) of which were verified as fake articles. From the GPT-4 model, 257 articles were fact-checked, 53 (20.6% [95% CI, 15.8%-26.1%]) of which were verified as fake articles (Table 2). The error rate of reference citing for GPT-4 was significantly lower than that for GPT-3.5 (*P* < .001) but remains nonnegligible. Narrower topics tended to have more fake articles than broader topics.

**Table 2.** Fake Journal Article References Cited by ChatGPT

Fact-checking	ChatGPT model	
	GPT-4	GPT-3.5
Total No. of articles checked	257	162
No. of fake articles	53	159
Error rate (95% CI), %^a^	20.6 (15.8-26.1)	98.1 (94.7-99.6)
Example of fake journal articles^b^	Kesselheim AS, Cresswell K. Implementing learning health systems in the UK NHS. *BMJ*. 2017; 357: j2449. doi:10.1136/bmj.j2449	Rubin JC et al. Building a learning health system: challenges and opportunities. *J Am Med Inform Assoc*. 2015.
	Niska R, Hane CA, Castillo RC. Development and validation of the XGBoost prediction model for stroke risk: a large-scale electronic health record-based cohort study. *J Stroke Cerebrovasc Dis*. 2018;27(9):2413-2422. doi:10.1016/j.jstrokecerebrovasdis.2018.04.010	Chen et al. Integrating patient graphs and knowledge graphs for lung cancer risk factor identification. *J Biomed Inform*. 2022.

^a^ Comparison of error rates between GPT-4 and GPT-3.5: *P* < .001.

^b^ Fake references were identified in the responses from ChatGPT about a large spectrum of topics in the learning health systems field. The latest GPT-4 (paid version) and the default GPT-3.5 (free version) were compared. Only 2 fake references are shown here as examples.

GPT-4 provided answers that could be used as supplementary materials for LHS training after fact-checking and editing. However, it failed to provide information about the latest LHS developments.

## Discussion

Our findings suggest that GPT-4 can be a helpful copilot in preparing new LHS education and training materials, although it may lack the latest information. Because GPT-4 cites some fake journal articles, they must be verified manually by humans; GPT-3.5–cited references should not be used.

When asked why it returned fake references, ChatGPT explained that the training data may be unreliable, or the model may not be able to distinguish between reliable and unreliable sources. As generative chatbots are deployed as copilots in health care education and training, understanding their unique abilities (eg, the ability to answer any questions) and inherent defects (eg, the inability to fact-check responses) will help make more effective use of the new GPT technology for improving health care education and training. Additionally, potential ethical issues such as misinformation and data bias should be considered for GPT applications.

This study has some limitations, such as the chat topics not representing all subject areas. However, since the LHS topics covered many subject areas of health care, the findings should be applicable in the health care domain. Furthermore, the findings should be more applicable to deeper discussions with ChatGPT as opposed to superficial discussions.

## Notes

Supplement.
Data Sharing Statement
