# Supplementary material for: Accuracy of Chatbots in Citing Journal Articles
Source: JAMA Netw Open. 2023 Aug 8;6(8):e2327647. doi: 10.1001/jamanetworkopen.2023.27647 (PMC10410472; doi:10.1001/jamanetworkopen.2023.27647)
Supplement: Supplement. — Data Sharing Statement [file jamanetwopen-e2327647-s001.pdf]

Data Sharing Statement

Chen. Accuracy of Chatbots in Citing Journal Articles. *JAMA Netw Open*. Published August 08, 2023. doi:10.1001/jamanetworkopen.2023.27647

Data
Data available: Yes
Data types: Data (not involving human participants)
How to access data: ajchen@web2express.org
When available: With publication

Supporting Documents
Document types: None

Additional Information
Who can access the data: Anyone with reasonable request.
Types of analyses: For any purpose.
Mechanisms of data availability: Without investigator support.
